# Long‐Range Hot‐Carrier Transport in Topologically Connected HgTe Quantum Dots

**DOI:** 10.1002/advs.202307396

**Published:** 2024-01-15

**Authors:** Xinning Huang, Yilu Qin, Tianle Guo, Jingjing Liu, Zhourui Hu, Jiale Shang, Hongfu Li, Gongrong Deng, Shuaiqin Wu, Yan Chen, Tie Lin, Hong Shen, Jun Ge, Xiangjian Meng, Xudong Wang, Junhao Chu, Jianlu Wang

**Affiliations:** ^1^ State Key Laboratory of Infrared Physics Shanghai Institute of Technical Physics Chinese Academy of Sciences 500 Yu Tian Road Shanghai 200083 China; ^2^ University of Chinese Academy of Sciences No. 19 A Yuquan Road Beijing 100049 China; ^3^ Hangzhou Institute for Advanced Study University of Chinese Academy of Sciences Chinese Academy of Sciences Hangzhou 330106 China; ^4^ Kunming Institute of Physics Kunming Yunnan 650223 China; ^5^ Frontier Institute of Chip and System Institute of Optoelectronics Shanghai Frontier Base of Intelligent Optoelectronics and Perception Fudan University Shanghai 200438 China

**Keywords:** colloidal quantum dots, hot‐carriers, honeycomb nanogeometry

## Abstract

The utilization of hot carriers as a means to surpass the Shockley‐Queasier limit represents a promising strategy for advancing highly efficient photovoltaic devices. Quantum dots, owing to their discrete energy states and limited multi‐phonon cooling process, are regarded as one of the most promising materials. However, in practical implementations, the presence of numerous defects and discontinuities in colloidal quantum dot (CQD) films significantly curtails the transport distance of hot carriers. In this study, the harnessing of excess energies from hot‐carriers is successfully demonstrated and a world‐record carrier diffusion length of 15 µm is observed for the first time in colloidal systems, surpassing existing hot‐carrier materials by more than tenfold. The observed phenomenon is attributed to the specifically designed honeycomb‐like topological structures in a HgTe CQD superlattice, with its long‐range periodicity confirmed by High‐Resolution Transmission Electron Microscopy(HR‐TEM), Selected Area Electron Diffraction(SAED) patterns, and low‐angle X‐ray diffraction (XRD). In such a superlattice, nonlocal hot carrier transport is supported by three unique physical properties: the wavelength‐independent responsivity, linear output characteristics and microsecond fast photoresponse. These findings underscore the potential of HgTe CQD superlattices as a feasible approach for efficient hot carrier collection, thereby paving the way for practical applications in highly sensitive photodetection and solar energy harvesting.

## Graphical abstract

A designed honeycomb‐like topological structures in the HgTe CQD superlattice introduced a long‐range hot carrier transport and microsecond fast photoresponse. Exhibiting the potential of HgTe CQD superlattices as a feasible approach for hot carrier collection, thereby paving the way for practical applications in highly efficient solar cells and sensitive photodetection.

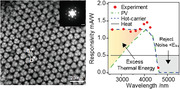

## 1 Introduction

The utilization of excess energy from hot‐carrier is thekey to overcome the Shockley–Queisser limit in photon‐electron conversion process,^[^ ^1^, ^2^ ^]^ such as solar energy collection, photodetection and etc. During the past decades, enormous efforts have been devoted to hunting efficient hot‐carrier extraction materials.^[^ ^3^, ^4^ ^]^ It was believed that spatially confined semiconductors, especially quantum dots are very promising because of their strong Coulomb interaction,^[^ ^5^ ^]^ high exciton binding energy,^[^ ^6^ ^]^ reduced charge screening and discrete energy states,^[^ ^7^ ^]^ leading to restricted multi‐phonon cooling processes and much longer hot‐carrier cooling time in prediction.^[^ ^8^ ^]^ However, due to the combination effects from inter and intra‐band Auger recombination^[^ ^9^ ^]^ and Auger‐like energy transfer^[^ ^10^ ^]^ in quantum dots, lifetimes of hot‐carriers were merely 10–100 ps,^[^ ^11^ ^]^ too short for charges’ collection. Therefore, the observation of hot‐carrier transport was reported only in a few quantum dot systems. In 2010, Tisdale and co‐workers^[^ ^12^ ^]^ showed the first observation of hot‐electron transfer from colloidal lead selenide (PbSe) nanocrystals to a titanium dioxide by time‐resolved optical second harmonic generation. And later in 2017, through 2D mapping of pump‐probe spectroscopy, a 600 nm transport distance before reaching the diffusive transport limit was reported in methylammonium lead iodide (CH_3_NH_3_PbI_3_) quantum dot films.^[^ ^13^ ^]^ Up to now, the hot‐carriers’ travel distances before they lose their excess energy to the lattice are still relatively low for practical applications.

Apart from slowed cooling process, another critical parameter to maximize hot‐carriers’ collection is carriers’ mean free path before scattering.^[^ ^14^ ^]^ In colloidal quantum dots system, there is a large number of defects^[^ ^15^, ^16^ ^]^ and discontinuities^[^ ^17^ ^]^ presented in CQDs films, which result in much enhanced scattering probabilities. One possible solution to enhance carriers' transport distance was to form a quasi‐3D superlattice by the CQDs as the artificial giant atoms.^[^ ^18^ ^]^ Such assemblies are predicted to possess unique electronic miniband structures,^[^ ^19^ ^]^ and consequently their electronic wavefunctions are likely overlapped and electrons being delocalized,^[^ ^19^, ^20^ ^]^ leading to the realization of carriers’ coherent transport or even edge state transport in which scattering could in principle be completely ignored.^[^ ^21^ ^]^ Thus, hot‐carriers of CQDs superlattices could have in theory traveled much further than a few nanocrystals and made it possible for practical device fabrications.

In this work, based on synthesized highly monodisperse HgTe CQDs, we have successfully created honeycomb‐like topologically connected CQDs superlattice, where its long‐range periodicity was confirmed by HR‐TEM, SAED patterns and low‐angle XRD. In such a system, a strong hot carrier assisted photocurrent was observed, and the utilization of excess hot electron energy from ≈4–2 Eg (2500–4300 nm) was proved by identical hot‐carrier's responsivity behavior in excitation energy dependent photocurrent measurements. In conjunction with unique linear output curves, micro‐second fast photoelectric conversion process and good Ohmic contacts, these findings strongly suggest the presence of nonlocal hot carrier transport, as opposed to a typical photovoltaic effect resulting from interface potential. Additionally, in a lateral HgTe CQD's architecture, we have obtained a striking long diffusion length (≈15 um) by 2D raster photocurrent mapping, surpassing existing hot carrier materials by more than tenfold.^[^ ^1^, ^22^, ^23^, ^24^ ^]^ Such an abnormal diffusion length could be an early indication that the light‐induced energy was transported away and collected through edge states, originating from topologically non‐trivial flat‐band in artificially assembled nanocrystals superlattice. This research provides an unexpected avenue for hot‐carrier collection materials, and may pave the way for practical applications of artificial molecular crystals that enable more efficient photodetection and solar energy harvesting.

**Figure 1:**
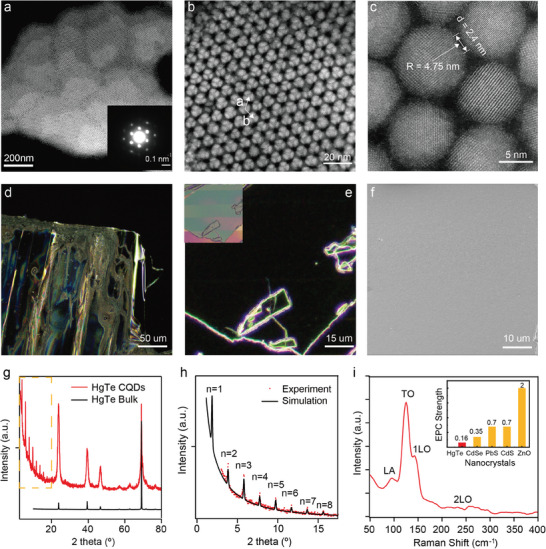
Characterization of morphology and crystal structure of HgTe quantum dots superlattice. a) HR‐TEM image of micro‐meter HgTe quantum dots pack. Inset: corresponding SAED images, scale bar: 0.1 nm^−1^. The lattices parameter of CQDs superlattice obtained from the 2‐set bright fingers was 9.5 nm, same as quantum dot's diameter, showing the long‐range ordering of the structure. b) High angle annular dark field scanning transmission electron microscopy (HAADF‐STEM) image of CQDs’ honeycomb structure (bright on a dark background). The lattice parameters a = b = 9.6 nm. c) High resolution HAADF‐STEM pictures resolve the atomic structure, outer shape and inter‐connection width d of the nanocrystals. d,e) Dark‐field optical micrograph of CQDs films on highly ordered pyrolytic graphite (HOPG) and silicon. For better illustration of film quality, superlattice on contacts with broken edges was picked. Here, almost no diffraction was observed inside CQDs superlattice. Inset: corresponding bright‐field optical images. f) Scanning electron microscope (SEM) image showing the surface of CQD superlattice. g) XRD patterns for HgTe CQDs superlattice. XRD peaks above 20° matched well with HgTe bulk powder reference. h) Zoomed‐in low‐angle XRD pattern shows periodic peaks, corresponding to the crystal parameters of superlattice (HgTe dots as artificial atoms). Order of Diffraction n was marked on top of each peak. i) Resonance Raman spectra of HgTe CQDs. The 2LO/1LO intensity ratio is ≈0.16, which represents the electron‐phonon coupling (EPC) strength of semiconductor nanocrystal. Inset: Comparison of EPC of several common nanocrystal. The 0.16 2LO/1LO ratio of HgTe CQDs was the lowest among all reported CQDs.

**Results and Discussion** To probe and maximize hot‐carrier transport in CQDs systems, it is ultimately inevitable to establish the large area connected quantum dots networks, in which most QDs are strongly coupled and homogenous for the entire channel of the test devices. Therefore, highly monodisperse, regular‐shape quantum dots with minimum size distribution became the building blocks of such systems.^[^ ^19^ ^]^ Here, we began by synthesizing crystalline HgTe quantum dots via a modification of a previous method^[^ ^25^ ^]^ using HgCl_2_ as Hg precursors and (TMS)_2_Te as Te precursors. In short, during conventional synthesis, 3 min after the hot injection of Te precursors, CQDs already passed the explosive nucleation stage and slowly grew into target sizes. At this point, we rapidly injected a low boiling point polar solvent (pure water) into the reaction. After the injection of water, abundant dynamic H_2_O/Oleylamine (Olam) interfaces (originating from the immiscibility between those two solvents) were generated under stirring and then a selective crystal‐oriented surface modification was conducted on surfaces of mercury quantum dots. As a result, particles subjected to additional water injection display enhanced monodispersity, accompanied by a much more spherical shape compared to those produced using typical hot‐injection methods (Figure S1, Supporting Information). As is shown in Figure S2 (Supporting Information), a 7% size distribution of mean sizes 9.6 nm was achieved in our HgTe quantum dots, better than most reported HgTe CQDs of similar sizes. Thus, the emergence of long‐range ordered quantum dots superstructures is expected and a well‐defined honeycomb‐like structure was clearly observed in TEM, shown in **Figure** **1**b and Figure S4 (Supporting Information). Here, we were able to distinctly observe a stacked superlattice structure, with half honeycomb rings of the first layer being prominently displayed, while those of the second layer appeared subdued. The structure of the superlattice was further confirmed by SAED analysis (Inset: Figure 1a). The patterns observed along the (001) axis displayed at least two orders of diffraction spots, which further proved the existence of long‐range periodicity over a scale larger than micrometers. Detailed discussion can be found in Figure S16 (Supporting Information).

In order to gain a comprehensive understanding of the fundamental properties of HgTe quantum dots superlattice, it is imperative to acquire thin films on substrate for detailed structural characterization. And additionally, as‐synthesized CQDs are usually insulating in a non‐polar solution (Hexane) with Olam as their capsulating ligands. In order to make them conducting and ready for opto‐electronic characterization, we have conducted liquid‐phase ligands exchange followed by almost the same protocol in an earlier publication.^[^ ^26^ ^]^ The only difference is that instead of Dimethylformamide, we chose 2‐6‐Difluoropyridine to be our target solution, in which a better solubility was obtained. As is shown in Figure 1c, the spherical outer shape of HgTe CQD was clearly presented, with 2.4 nm inter‐connection width d of the nanocrystals. In the film preparation, we are trying to preserve the long‐range periodicity observed earlier in TEM images. In some earlier reports long‐range periodicity was related to unique physical phenomena, such as the band‐like transport mechanism in CQDs films,^[^ ^27^, ^28^ ^]^ coherent excitons behavior (super‐radiance) in perovskite superlattice,^[^ ^29^ ^]^ the emergence of topologically non‐trivial flat band^[^ ^30^ ^]^ and etc. Therefore, in order to observe long‐range hot‐carrier transport in colloidal quantum dots systems, it would be necessary to fabricate the films into topologically connected quantum dots superlattice, due to the much stronger coupling strength in‐between carriers. Traditionally,^[^ ^31^ ^]^ such colloidal quantum dots superlattice was mostly achieved by controlling the solvent evaporation that allows the low‐rate self‐assembly of QDs from bulk colloids into denser solid building blocks. However, in this work, before the slow evaporation for self‐assembly, we employed a blade coating procedure to form a uniform layer which is similar to a “seed crystal” for later assembly. During the coating process, our HgTe CQDs ink was first deposited on a processed silicon blade edge to form a meniscus between the substrate and the blade edge. Then, the blade is subsequently dragged over the substrate at a high speed (30 mm s^−1^) to enable film formation in the Landu–Levich regime. After the blade stopped at the end of silicon substrate, the blade was lifted out of the quantum dot solution, leaving them for slow evaporation in a closed box, eventually forming a sub‐micron thick film with a mirror‐like surface. As illustrated in the dark field optical images presented in Figure 1d,e, we were able to obtain hundreds of micrometer‐sized CQDs flakes that almost no observable defects under 100x objective lens, which is also confirmed through SEM (shown in Figure 1f). Moreover, to evaluate the crystallinity of our CQDs films, XRD experiments were conducted. As is shown in Figure 1g, apart from typical HgTe crystal lattice information, we have also observed a series of sharp peaks at small angles (2θ = 3.87^o^, 5.79^o^, 7.78^o^ etc.). Those periodic peaks (spacing Δ_2θ_ = 1.96°) reflected the first and high‐order of diffraction from structured HgTe quantum dots as artificial atoms. The estimated first‐order peak (2θ = 1.86^o^) reflects lattice parameters of 4.75 nm (half of HgTe CQD's size), indicating an A‐B stacking structure. Detailed simulation and discussion can be found in Supporting Information.

**Figure 2:**
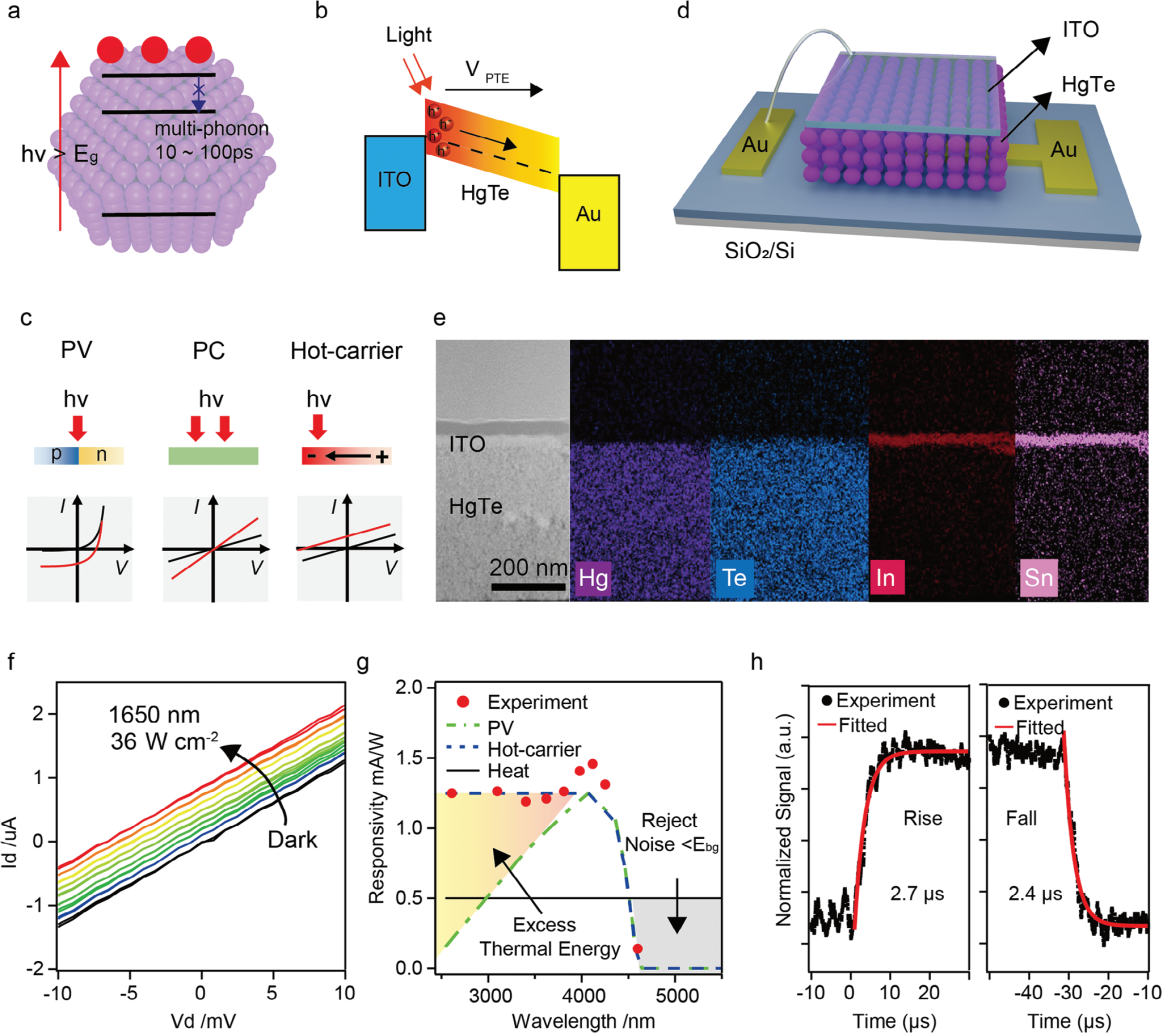
Hot‐carrier assisted photo‐electron conversion. a) Schematic illustration of hot‐carriers generation and recombination. b) Schematic diagram of directional flow of electrons caused by temperature gradient. c) Comparison of three different light‐to‐current conversion mechanisms. Black and red lines illustrate typical *I–V* characteristics in the dark and under illumination, respectively. d) Schematic diagram of vertical CQD superlattice device. e) Cross‐section TEM image of the HgTe/ITO interface and EDS mapping, where the elements Hg, Te, In, and Sn are marked with different colors, showing no obvious interdiffusion layer between HgTe CQD and ITO. f) Output *I–V* curve of CQD‐based hot‐carrier device under 1650 nm laser illumination. Laser power density was increased from zero to the maximum of 36 W/cm^2^. g) Illustration of relative spectral response based different mechanism, PV, hot‐carrier and heat. Experiment results (red dots) shown the responsivity as functions of wavelength at a fix incident power intensity of 1.4 W cm^−2^ and zero bias, relatively no responsivity changes with changed laser wavelength until the band edge. h) Rise and fall times of the normalized signal under zero bias and at 1650 nm laser. Demonstrating a fast response time less than 3 µs.

In the detection and identification of hot‐carrier transport, it is important to understand the evolution and unique behaviors of charge carriers in photon‐electron conversion, as is illustrated in **Figure** **2**a. Upon illumination with an above‐bandgap laser, charge carriers are created at the elevated positions (within a short time‐frame ≈10 fs), hot carriers would successively decrease their excess energy through phonon interactions and eventually establish a thermal equilibrium with the lattice. However, those electron‐phonon interactions would be delayed in CQDs due to restricted multi‐phonon processes between quantum‐confined discrete energy states. In colloidal quantum dots, such time would be increased to almost 100 ps, long enough to probe and collect some of hot‐carriers. Here, the weaker electron‐phonon interaction could be Quasi‐quantitatively represented by the intensity ratio of the second harmonic to the fundamental, proposed by Kelley et al^[^ ^32^ ^]^ in a series of earlier works. This ratio is used to estimate Huang–Rhys parameter (S), which is directly linked to the strength of the electron‐phonon or exciton‐phonon coupling. As is observed in Figure 1i, our 2LO/LO intensity ratio of HgTe film was merely 0.16, one of the lowest in quantum confined systems,^[^ ^33^ ^]^ indicating a fairly weak electron‐phonon interaction. Combined with minimized defects in superlattice, it was one step closer to observe long‐range hot‐carrier transport.

To observe hot carrier's transport electronically, it was necessary to form a net current flow. As is shown in Figure 2b, due to slowed cooling process, a carrier temperature gradient would have been created between the excitation hot region and the far end (cool region) upon illumination. Thus, the photocurrent is therefore generated by diffusion of those hot carriers, with a rather unique linear output curve shift upward/downward with light illumination, as is shown in Figure 2c. For comparison, detectors based on photovoltaic effect would have a nonlinear current–voltage (*I‐*‐*V*) curve with a short‐circuit current, while those based on photoconductive effect works in a linear *I‐*‐*V* curve (conductivity increased under illumination).

In an effort to gain further insight into the mechanism of photoinduced charge carriers, a vertical transparent electrodes‐CQDs‐metal electrodes structure (Figure 2d) was fabricated through micro‐fabrication techniques. It was believed that a good interface was formed with minor damage on HgTe function layer, as was evidenced by cross‐sectional TEM image and energy‐dispersive X‐ray spectrometry (EDS) mapping (shown in Figure 2e), where components of Hg, Te, In and Sn are dense and clearly identified and marked with different colors, in which, no interdiffusion had been observed between ITO and HgTe CQDs films. We then performed the photocurrent measurements with the illumination of 1650 nm lasers at 36 W cm^−2^ power. For those devices based on conventional synthesized HgTe CQDs films, we have observed typical photoconductive response in the 10 µm × 10 µm × 500 nm vertical structure pixels (Figure S9, Supporting Information). However, in those devices based on HgTe CQDs superlattice, we have successfully observed the typical linear *I–V* output curves with upward shifted line under continuous light illumination, as is shown in Figure 2f. Here, a net current flowing from the excitation to the far end was observed, indicating the p‐type nature of our HgTe CQDs, which is consistent with Seebeck measurements in Figure S10 (Supporting Information). The voltage generated in the device is ≈−1 µA or 7 mV at the max power, which corresponds to a responsivity of ∼1 mA W^−1^ or 35 V W^−1^, higher than most of the 2D materials (e.g., Graphene,^[^ ^34^, ^35^, ^36^ ^]^ MoS_2_,^[^ ^37^ ^]^ Black phosphorus).^[^ ^38^ ^]^ Here, based on the Seebeck coefficient we got (Figure S10, Supporting Information), the temperature gradient of hot‐carrier is estimated to be ≈35 K µm^−1^, if assuming all energy from elevated hot carrier was collected. However, it was obviously inaccurate because with our 36 W cm^−2^ laser, we are expected to heat the electron/holes into 1200 K, which is 2400 K µm^−1^, ≈100‐fold higher than what we observed. Thus, in a rough estimation, the electron‐phonon cooling time should be ≈1/100 to the speed of carrier collection through electrodes. As a result, if electron‐phonon interaction was further delayed by other modifications (such as additional shell layer, plasmon enhancement, etc.), we believed the conversion efficiency could be greatly improved. Besides, we employed the structure (Figure 2d) to exhibit a potential to be the photodetector, as shown in Figures S17 and S18 (Supporting Information).

Apart from typical linear *I–V* output, another identical phenomenon in hot‐carrier devices was the energy independent responsivity. As is shown in Figure 2g, we have conducted the measurements of responsivity in a function of incident light wavelength with fixed power at ≈1.4 W cm^−2^. In conventional photovoltaic devices, it exhibits a distinctive wavelength‐dependent response per unit of incident radiation power. This response is directly related to the arrival rate of photons, considering that the energy carried by each photon is inversely proportional to the wavelength. Consequently, the spectral response progressively increases as the wavelength extends, until it reaches the cutoff wavelength determined by the detector material. The cutoff wavelength denotes the point at which the detector's responsivity declines to 50% of its peak responsivity. In contrast, hot‐carriers’ devices do not rely on the photonic nature of the incident radiation. Because the response solely depends on the radiant power or its rate of change, rather than the spectral content, the response of our detectors to radiation remains independent of the wavelength before reaching the cutoff wavelength (those little fluctuations observed were attributed to minor variations in laser power). We have also considered the possibility of ununiform light absorption, and plotted the absorption spectra of HgTe CQDs superlattices, in which a relatively uniform spectral responsivity can be observed in Figure S6 (Supporting Information). Besides, it is important to note that carrier multiplication, if it were to occur, would only happen in the wavelength range before 2.07 µm, as the absorption cutoff of the quantum dots is at 4.15 µm. In an ideal case, carrier multiplication would require incident photons to have at least twice the energy of the bandgap energy. However, this is not the case in reality, as shown in Figure 2g, where the responsivity remains almost constant before the absorption cutoff, indicating the absence of carrier multiplication at current wavelength range. With the confirmation of energy independent photoresponse, it should be safe to claim that we indeed observed a thermoelectric behavior that utilizes excess thermo‐energy of charge carriers in HgTe CQDs superlattice. To further verify the assistance of hot‐carriers, it is necessary to acquire the speed of photoresponse in the devices. As is described in earlier section, conventional thermoelectric devices were based on phonon interaction, in which the generation, transportation and collection are all in the time scale of milliseconds, sometimes even seconds. However, with a SR560 preamplifier and oscilloscope, a micro‐second fast photo response was observed, as is shown in Figure 2h. The rise and fall time were both lower than 3 µs, limited by the bandwidth of our amplifier. To sum up, with the combination of unique linear photoresponse output curve, wavelength independent responsivity and micro‐second fast response time, it was believed that the self‐driven photocurrent was indeed coming from hot‐carriers.

**Figure 3:**
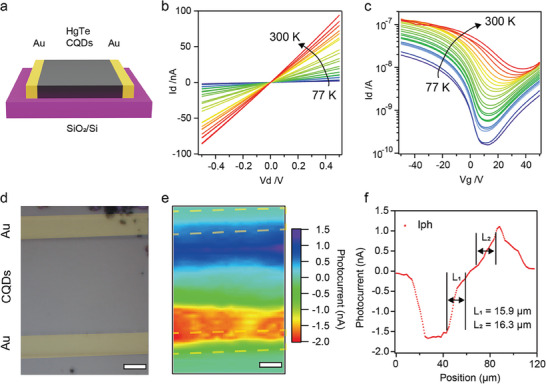
Electrical properties of HgTe CQDs superlattice and Long‐range Hot‐carrier collection. a) Schematic diagram of lateral device. b,c) electrical properties of HgTe superlattice. Output curves and transfer curves of p‐type on CQD solids from 77 to 300 K. d–f) Photocurrent mapping measured at zero bias in 1650 nm laser illumination. d) Optical image of the measured device. Scale bar, 10 µm. e) Photocurrent mapping images. f) Extracted *I*_ph_ along the channel.

**Figure 4:**
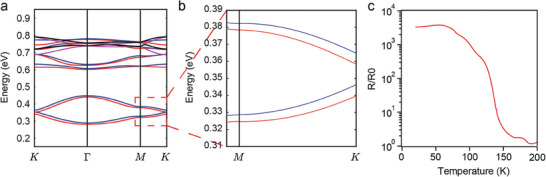
Simulation and Experimental indication of Topological Edge States. a) Conduction band dispersions resulting from the atomistic tight‐binding (TB) calculation for D = 9.5 nm and d/D = 0.5. b) zoomed‐in band diagram near K point. c) Resistivity measurement from 10 to 200 K. Here, R0 is the resistivity at room temperature.

Before we conduct further experiments on transport mechanism, we have observed an unexpected trend during the photocurrent measurements in vertical structure devices. As illustrated in Figures S11 and S12 (Supporting Information), we noticed that the thicker the CQDs superlattice, the higher hot‐carrier assisted photo‐current and the highest 18 mV photo‐induced voltage was observed in the thickest 750 nm film with no indication of saturation at all. The 750 nm distance was significantly higher than our expectation based on typical CQDs’ mobility and hot‐carrier cooling time. Therefore, to quantify intrinsic electrical properties of our HgTe films, we have fabricated devices in a lateral architecture of field‐effect transistor, as is illustrated in **Figure** **3**a. First, to rule out the possibilities of any interfacial Schottky junction, we obtained the output characteristic curves without any illumination, in which a good linear relationship was kept from 77 to 300 K (Figure 3b). In conjugation with nanometer depletion layer width of simulated Schottky junction and less than 100 nm diffusion lengths in conventional HgTe CQDs films, we believed there is a negligible contribution from Schottky junction in observed hot‐carrier assisted photocurrent (Details in Supporting Information). Besides the corresponding transfer characteristic curves reveal that our quantum dots are of slightly p‐type, and our mobility was calculated to be 0.34 cm^2^V^−1^S^−1^ at 300 K (Figure 3c). Strangely, at current mobility level and a 100 ps cooling time, a diffusion length of less than 300 nm was expected. To better quantify the actual diffusion length in our CQDs superlattice, we have conducted a 2D raster mapping under illumination of a 1650 nm laser at zero bias (Figure 3d–f). The corresponding optical image is shown in **Figure** **4**a and our channel and electrodes are remarked with gray and golden yellow. In conventional HgTe CQDs films, the photo‐active area was almost fully‐confined in gold electrodes, as is shown in Figure S14 (Supporting Information). However, in HgTe CQDs superlattice, we have observed a striking broad photo‐active area in the results of photocurrent mapping and the extracted diffusion length was ≈15 µm, as is shown in Figure 3e,f. Such an enormously long diffusion length with no external driven force was in consist with enhanced photocurrent at thicker films and indicated the existence of an additional transport mechanism beyond simple carriers’ diffusion.

One possible explanation was that in such specifically formed honeycomb nanogeometry films, electrons moved in strongly correlated states without encountering conventional diffractions from defects. In earlier work from D. Vanmaekelbergh and C. Morais Smith,^[^ ^39^ ^]^ they proposed that topological non‐trivial flat bands would be generated through the combination effect of strong spin‐orbit coupling of HgTe and the honeycomb structure. Here, as is shown in Figure 4a,b, applying similar atomistic TB calculations with our superlattice parameters, we have found the existence of flat bands that were protected by a gap close to 30 meV. In addition, when we conducted electrical transport measurements (Figure 4c), unlike most conventional colloidal quantum dots, the conductivity entered a plateau at below 80 K instead of exponentially dropping. In other word, our HgTe superlattice, exhibits an insulating behavior (attributed to the bulk conductivity) at high *T* and a saturating and weakly metallic behavior (attributed to the residual surface conduction after bulk carriers freeze out) at low *T*. Therefore, the observed behavior might become an early indication of the existence of topological edge states.

**Conclusion** In conclusion, we present a novel topologically connected HgTe colloidal quantum dots superlattice. The long‐range periodicity of superstructures was verified through a series of characterization techniques. With a simple vertical device design, fast and efficient hot‐carrier assisted photo‐electron conversion has been demonstrated by a combination of energy independent responsivity, linear output characteristic and micro‐second fast photoresponse. In addition, through 2D raster mapping under illumination of a 1650 nm laser at zero bias, we have made the first observation of long hot‐carrier transport within colloidal quantum dots themselves. The ≈15 µm hot‐carrier transport length exceeds those of any previously reported materials, indicating the existence of a more complex transport mechanism beyond simple carrier diffusion. Our results provide the possibility of utilizing hot‐carriers in practical photodetectors and solar cells with efficiencies beyond the thermodynamic limit.

## 2 Experimental Section

The synthetic methods of HgTe CQDs are provided in the Supporting Information.

### Instrumentation and Materials Characterization

To characterize the materials, SEM (Zeiss Ultra 55, operating at 5.0 kV), TEM (JEM‐2011, operating at 80, 120 or 200 kV), Spherical Aberration Corrected Transmission Electron Microscope (JEM‐ARM200F, HAADF‐STEM mode), AFM (Oxford Cypher), optical microscopy (Leica DM4000M) were used. XRD was done on a Bruker D8 Discover diffractometer operating with a Cu K α energy source at 40 kV and 40 mA was bestowed to crystallographically characterize HgTe CQDs. The optical absorption spectra were collected using a Thermofisher Nicolet iS50 Fourier Transform Infrared. The electron‐phonon coupling strength was carried out by Resonance Raman Spectroscopy with the Thermofisher DXR2i. The cross‐section sample of ITO‐HgTe CQDs‐metal sandwich photodetectors was treated by using a focused ion beam (FIB) lift‐out technique with Tescan GAIA3. After the FIB process, the TEM images and EDS results were obtained by using Thermofisher TALOS F200X. More parameters used in the characterizations are included in the Supporting Information.

### Device Fabrication

The source and drain electrode patterns were fabricated by standard ultraviolet lithography. Titanium/gold (Ti/Au), 15/45 nm electrodes were deposited by electron beam evaporation. Then, a 500 nm‐thick HgTe CQDS film was blade‐coated on the fabricated SiO2/Si substrates with patterned electrodes (heated at 45 °C). Then a 70 nm ITO film for the transparent top electrode was deposited by dual ion beam sputtering. Therefore, the metal‐HgTe CQDs‐metal sandwich photodetectors were successfully fabricated.

### Photocurrent Measurements

All the optoelectronic measurements were taken by Mstarter 200 optoelectronic measurement system from Matia Optoelectronic Technology Co., LTD, at room temperature in ambient conditions. High time‐resolution response current signal was converted to a voltage signal using a preamp (Stanford Research Systems SR560) and recorded by Tektronix MDO3014 mixed domain oscilloscope.

### Temperature‐Dependent Conductivity Measurements

In the temperature‐dependent conductivity (from 12 to 300 K) measurements, the standard four‐probe method was used in a Lake Shore Cryotronics system under vacuum. In the system, a sapphire plate (Al_2_O_3_) was used to anchor the samples to the copper holder; the samples were attached to the sapphire plate with a Dow Corning silicone heat sink compound. Electrical contacts were made with tungsten probes and the drain was connected to the ground. Electrical measurements were conducted by Keysight B1500A, which measures the voltage drop with varying applied currents. The conductivity of the four‐contact measurements was calculated through the following equation:

(1) $$\sigma =\frac{dI}{dV}*\frac{L}{W*t}$$

where dI/dV is the slope of the *I–V* curve, W is the width of the sample, t is the thickness of the flake, and L is the distance between the two voltage probes. For temperature‐dependent conductivity measurement, the sample was first cooled to 12 K, and the conductivities at different temperatures were recorded after the temperature was reached and stabilized for 15 min.

## Conflict of Interest

The authors declare no conflict of interest.

## Author Contributions

X.H. and Y.Q. contributed equally to this work. T.G., J.L. and J.W. designed the research. T.G., J.L., Z.H. performed the material preparation, X.H., Y.Q. and T.G. performed the analysis of the mechanism, designed and fabricated the electrical devices and most regular characterizations. J.G., H.L. and G.D. performed infrared photoresponsivity experiments. X.H. and J.S. performed all the simulations. S.W., Y.C., X.M. and T.L. discussed the charge transport mechanism. H.S. and X.W. gave important advises of device fabrication to improve hot‐carriers’ collection efficiency. J.W., J.L. and T.G. supervised the research. J.W. and J.C. provided many insightful remarks and suggestions. J.L. and T.G. wrote the draft of the paper. All authors discussed the results and commented on the manuscript.

## Supporting information

Supporting Information

## Data Availability

The data that support the findings of this study are available from the corresponding author upon reasonable request.

## References

[1] M. Li , J. Fu , Q. Xu , T. C. Sum , Adv. Mater. 2019, 31, 1802486. 10.1002/adma.201802486 30600555

[2] K. K. Paul , J.‐H. Kim , Y. H. Lee , Nat Rev Phys 2021, 3, 178.

[3] Y. Zheng , X. Wu , R. Zhuang , C. Tian , A. Sun , C. Tang , Y. Liu , Y. Hua , C. C. Chen , Adv. Funct. Mater. 2023, 33, 2300576.

[4] I. Nadinov , K. Almasabi , L. Gutiérrez‐Arzaluz , S. Thomas , B. E. Hasanov , O. M. Bakr , H. N. Alshareef , O. F. Mohammed , ACS Central Science 2023. 10.1021/acscentsci.3c00562 PMC10823510 38292602

[5] C. R. Kagan , E. Lifshitz , E. H. Sargent , D. V. Talapin , Science 2016, 353, aac5523. 27563099 10.1126/science.aac5523

[6] P. Kambhampati , Acc. Chem. Res. 2011, 44, 1. 20942416 10.1021/ar1000428

[7] Y.‐S. Park , J. Roh , B. T. Diroll , R. D. Schaller , V. I. Klimov , Nat. Rev. Mater. 2021, 6, 382.

[8] M. Li , S. Bhaumik , T. W. Goh , M. S. Kumar , N. Yantara , M. Grätzel , S. Mhaisalkar , N. Mathews , T. C. Sum , Nat. Commun. 2017, 8, 14350. 28176882 10.1038/ncomms14350 PMC5309769

[9] B. L. Wehrenberg , C. Wang , P. Guyot‐Sionnest , J Phys Chem B 2002, 106, 10634.

[10] R. Singh , W. Liu , J. Lim , I. Robel , V. I. Klimov , Nat. Nanotechnol. 2019, 14, 1035. 31591527 10.1038/s41565-019-0548-1

[11] H. Zhu , N. Song , T. Lian , J. Am. Chem. Soc. 2011, 133, 8762. 21534569 10.1021/ja202752s

[12] K. J. W. William , A. Tisdale , B. A. Timp , D. J. Norris , E. S. Aydil , X.‐Y. Zhu , Science 2010, 328, 1543. 20558714 10.1126/science.1185509

[13] Z. Guo , Y. Wan , M. Yang , J. Snaider , K. Zhu , L. Huang , Science 2017, 356, 59. 28386007 10.1126/science.aam7744

[14] S. K. Bux , R. G. Blair , P. K. Gogna , H. Lee , G. Chen , M. S. Dresselhaus , R. B. Kaner , J.‐P. Fleurial , Adv. Funct. Mater. 2009, 19, 2445.

[15] Y. Kobayashi , T. Nishimura , H. Yamaguchi , N. Tamai , J. Phys. Chem. Lett. 2011, 2, 1051.

[16] Y. Di , K. Ba , Y. Chen , X. Wang , M. Zhang , X. Huang , Y. Long , M. Liu , S. Zhang , W. Tang , Z. Huang , T. Lin , H. Shen , X. Meng , M. Han , Q. Liu , J. Wang , Adv. Sci. 2023, 2307169. 10.1002/advs.202307169 PMC10853715 38044286

[17] L. Gao , C. Chen , K. Zeng , C. Ge , D. Yang , H. Song , J. Tang , Light Sci Appl 2016, 5, e16126. 30167178 10.1038/lsa.2016.126 PMC6059941

[18] J. Pinna , R. Mehrabi Koushki , D. S. Gavhane , M. Ahmadi , S. Mutalik , M. Zohaib , L. Protesescu , B. J. Kooi , G. Portale , M. A. Loi , Adv. Mater. 2023, 35, 2207364. 10.1002/adma.202207364 36308048

[19] K. Whitham , J. Yang , B. H. Savitzky , L. F. Kourkoutis , F. Wise , T. Hanrath , Nat. Mater. 2016, 15, 557. 26901512 10.1038/nmat4576

[20] C. P. Collier , R. J. Saykally , J. J. Shiang , S. E. Henrichs , J. R. Heath , Science 1997, 277, 1978.

[21] C. Melnychuk , P. Guyot‐Sionnest , Chem. Rev. 2021, 121, 2325. 33428388 10.1021/acs.chemrev.0c00931

[22] S. Kahmann , M. A. Loi , J. Mater. Chem. C 2019, 7, 2471.

[23] J. Sung , C. Schnedermann , L. Ni , A. Sadhanala , R. Y. S. Chen , C. Cho , L. Priest , J. M. Lim , H.‐K. Kim , B. Monserrat , P. Kukura , A. Rao , Nat. Phys. 2019, 16, 171.

[24] C. Schnedermann , J. Sung , R. Pandya , S. D. Verma , R. Y. S. Chen , N. Gauriot , H. M. Bretscher , P. Kukura , A. Rao , J. Phys. Chem. Lett. 2019, 10, 6727. 31592672 10.1021/acs.jpclett.9b02437 PMC6844127

[25] G. Shen , M. Chen , P. Guyot‐Sionnest , J. Phys. Chem. Lett. 2017, 8, 2224. 28467091 10.1021/acs.jpclett.7b00775

[26] X. Xue , M. Chen , Y. Luo , T. Qin , X. Tang , Q. Hao , Light Sci Appl 2023, 12, 2. 36587039 10.1038/s41377-022-01014-0 PMC9805449

[27] X. Lan , M. Chen , M. H. Hudson , V. Kamysbayev , Y. Wang , P. Guyot‐Sionnest , D. V. Talapin , Nat. Mater. 2020, 19, 323. 31988516 10.1038/s41563-019-0582-2

[28] J.‐S. Lee , M. V. Kovalenko , J. Huang , D. S. Chung , D. V. Talapin , Nat. Nanotechnol. 2011, 6, 348. 21516091 10.1038/nnano.2011.46

[29] G. Rainò , M. A. Becker , M. I. Bodnarchuk , R. F. Mahrt , M. V. Kovalenko , T. Stöferle , Nature 2018, 563, 671. 30405237 10.1038/s41586-018-0683-0

[30] Z. Jiang , Z. Liu , H. Ma , W. Xia , Z. Liu , J. Liu , S. Cho , Y. Yang , J. Ding , J. Liu , Z. Huang , Y. Qiao , J. Shen , W. Jing , X. Liu , J. Liu , Y. Guo , D. Shen , Nat. Commun. 2023, 14, 4892. 37580381 10.1038/s41467-023-40515-3 PMC10425367

[31] M. C. Weidman , D.‐M. Smilgies , W. A. Tisdale , Nat. Mater. 2016, 15, 775. 26998914 10.1038/nmat4600

[32] K. Gong , D. F. Kelley , A. M. Kelley , J. Phys. Chem. C 2016, 120, 29533.

[33] A. M. Kelley , J. Chem. Phys. 2019, 151, 140901. 31615241 10.1063/1.5125147

[34] X. Cai , A. B. Sushkov , R. J. Suess , M. M. Jadidi , G. S. Jenkins , L. O. Nyakiti , R. L. Myers‐Ward , S. Li , J. Yan , D. K. Gaskill , T. E. Murphy , H. D. Drew , M. S. Fuhrer , Nat. Nanotechnol. 2014, 9, 814. 25194945 10.1038/nnano.2014.182

[35] A. L. Hsu , P. K. Herring , N. M. Gabor , S. Ha , Y. C. Shin , Y. Song , M. Chin , M. Dubey , A. P. Chandrakasan , J. Kong , P. Jarillo‐Herrero , T. Palacios , Nano Lett. 2015, 15, 7211. 26468687 10.1021/acs.nanolett.5b01755

[36] J. Tong , M. Muthee , S.‐Y. Chen , S. K. Yngvesson , J. Yan , Nano Lett. 2015, 15, 5295. 26218887 10.1021/acs.nanolett.5b01635

[37] M. Buscema , M. Barkelid , V. Zwiller , H. S. J. Van Der Zant , G. A. Steele , A. Castellanos‐Gomez , Nano Lett. 2013, 13, 358. 23301811 10.1021/nl303321g

[38] L. Viti , J. Hu , D. Coquillat , W. Knap , A. Tredicucci , A. Politano , M. S. Vitiello , Adv. Mater. 2015, 27, 5567. 26270791 10.1002/adma.201502052

[39] W. Beugeling , E. Kalesaki , C. Delerue , Y.‐M. Niquet , D. Vanmaekelbergh , C. M. Smith , Nat. Commun. 2015, 6, 6316. 25754462 10.1038/ncomms7316 PMC4366513

