# Supplementary material for: Long‐Range Hot‐Carrier Transport in Topologically Connected HgTe Quantum Dots
Source: Adv Sci (Weinh). 2024 Jan 15;11(12):2307396. doi: 10.1002/advs.202307396 (PMC10966574; doi:10.1002/advs.202307396)
Supplement: Supplementary file 1 — Supporting Information [file ADVS-11-2307396-s001.pdf]

ADVANCED SCIENCE
Open Access

# Supporting Information

for *Adv. Sci.*, DOI 10.1002/advs.202307396

Long-Range Hot-Carrier Transport in Topologically Connected HgTe Quantum Dots

*Xinning Huang, Yilu Qin, Tianle Guo*, Jingjing Liu*, Zhourui Hu, Jiale Shang, Hongfu Li, Gongrong Deng, Shuaiqin Wu, Yan Chen, Tie Lin, Hong Shen, Jun Ge, Xiangjian Meng, Xudong Wang, Junhao Chu and Jianlu Wang**

# Supplementary Information

# Long-range Hot-carrier transport in Topologically connected HgTe Quantum Dots

*Xinning Huang[1,2‡], Yilu Qin[1‡], Tianle Guo[1*], Jingjing Liu[1*], Zhourui Hu[1,4], Jiale Shang[1,2], Hongfu Li[5], Gongrong Deng[5], Shuaiqin Wu[1,3], Yan Chen[1,3], Tie Lin[1], Hong Shen[1], Jun Ge[1], Xiangjian Meng[1,2], Xudong Wang[1], Junhao Chu[1,2], Jianlu Wang[1,2,3, 4*]*

[1]State Key Laboratory of Infrared Physics, Shanghai Institute of Technical Physics, Chinese Academy of Sciences, 500 Yu Tian Road, Shanghai 200083, China.
[2]University of Chinese Academy of Sciences, No. 19 A Yuquan Road, Beijing 100049, China.
[3]Frontier Institute of Chip and System, Institute of Optoelectronics, Shanghai Frontier Base of Intelligent Optoelectronics and Perception, Fudan University, Shanghai 200438, China
[4]Hangzhou Institute for Advanced Study, University of Chinese Academy of Sciences, Chinese Academy of Sciences, Hangzhou 330106, China
[5]Kunming Institute of Physics, Kunming City, Yunnan Province, 650223, China
[‡] These authors contributed equally to this work
*Correspondence: guotianle@mail.sitp.ac.cn (T. G.), liujingjing@mail.sitp.ac.cn (J. L.), jlwang@mail.sitp.ac.cn (J. W.)

KEYWORDS. Hot-carriers, Colloidal Quantum Dots, Honeycomb nanogeometry.

**Contents:**

Supplementary materials and methods.
Supplementary Figures S1 – S18
Table S1

**Supplementary Materials and Methods.**

*Chemicals*

Mercury chloride ($HgCl_2$, Sigma-Aldrich, ≥99.5%), **mercury compounds are highly toxic. Handle them with special care.** Bis(trimethylsilyl)telluride ($(TMS)_2Te$, Fisher 98%), Oleylamine (OLA, Adamas, 90%), 1-dodecanethiol (DDT, Adamas, 98%), ethanol absolute anhydrous (Greagent, ≥99.7%), isopropanol (IPA, Greagent, ≥ 99.8%), hexane (Adamas, 99%), tetrachloroethylene(TCE, Adamas, 99%), Tetrabutylammonium chloride(Adamas, 95%), 2-mercaptoethanol(Sigma-Aldrich, ≥ 99.8%), n-butylamine (Adamas, 99%), N,N-dimethylformamide (DMF, Adamas, 99.8%+), 2,6-Difluoropyridine (DFP, Adamas, 98%+)

*Synthesize of HgTe quantum dots*

The HgTe CQDs were synthesized following a protocol by Shen et al[1]. Briefly, 54 mg (0.1 mmol) $HgCl_2$ were dissolved in 4 mL dried oleylamine at 100 °C under inert Ar. A mixture of 29 µL (0.05 mmol) of bis (trimethylsilyl) telluride in 0.3 mL anhydrous hexane was injected into the $Hg^{2+}$ containing solution. 3 mins after the injection of $(TMS)_2Te$, 1 ml of pure water (18 MΩ) was rapidly injected into the mixed system and stirred, and continued for the reaction. Then, after 30 mins of CQDs growth at 100 °C, the dispersion was quenched in 4 mL tetrachloroethylene. The CQDs were precipitated from the dispersion by adding 24 mL ethyl alcohol, centrifuged at 11 000 rpm for 1 min. the precipitate was re-dispersed in 5 mL Hexane and ready for ligands exchange, which is on the basis of a previously reported method. In a typical process, 140 mg tetrabutylammonium chloride, 70 µl 2-mercaptoethanol, 500 µl dodecanethiol

and 400 μl n-butylamine were dissolved in 5ml DMF, forming the hybrid ligand solution. Shaking vigorously for 1 min resulted in the transfer of HgTe QDs from hexane to DMF phase. Those CQDs were then precipitated by adding ethanol as the anti-solvent, followed by centrifugation at 7,000 r.p.m. for 60 second. After discarding the supernatant, 300 μl DFP was used to dissolve the HgTe QD solids and ready for blade-coating.

*TEM images of conventional and dynamic interface synthesis*

As is mentioned in the main text, by introducing water during the synthesis of HgTe colloidal quantum dots (CQDs), a notable improvement in the uniformity of individual quantum dot morphologies and an enhanced overall monodispersity were achieved, as is illustrated in following Fig. S1.

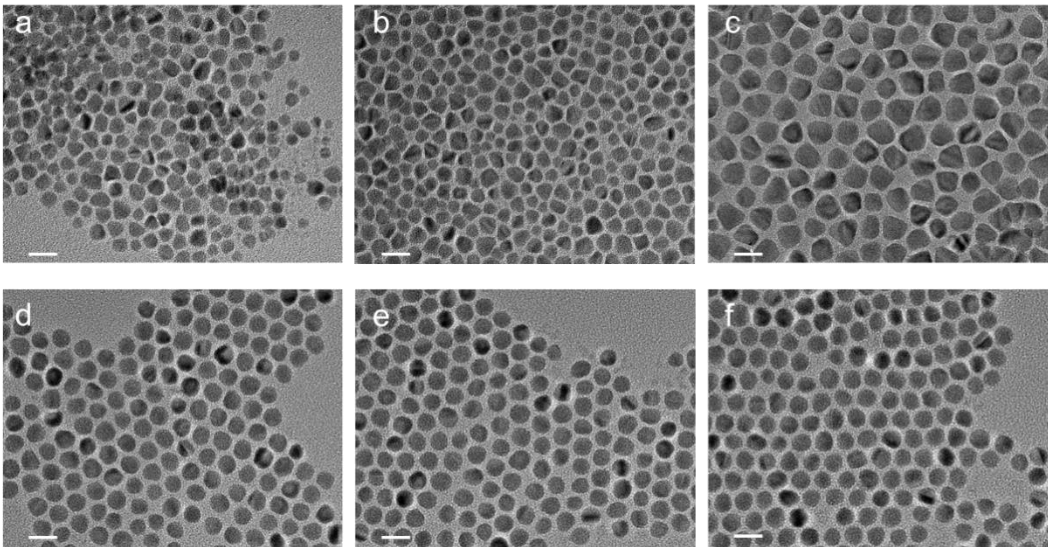


**Figure S1. TEM image of the HgTe CQDs. a-c)** Typical hot-injection methods. **d-e)** The hot-injection method with water. The scale bar is 20 nm.

*Mean size of HgTe CQDs by dynamic interface synthesis*

As is mentioned in main text, high quality HgTe CQDs were synthesized in our work. Here, more TEM images are provided, in Fig. S2 – Fig.S4. TEM images were analyzed manually and its statistic is summarized through histogram and fitted with a gaussian function which represents the size distribution. The volume is calculated using the mean radius/apex to base distance assuming they are perfect sphere. HgTe CQDs are reasonably modeled as a high-symmetry near-sphere as shown in Fig. S2. A mean size of our HgTe CQDs was about 9.6 nm (corresponding to 4.15 um cut-off absorption, shown in Figure S2), with a size distribution smaller than 7%, as shown in Figure S2. Such a quality is close to the state-of-art PbS quantum dots synthesis, whose size variation is usually less than 3%.

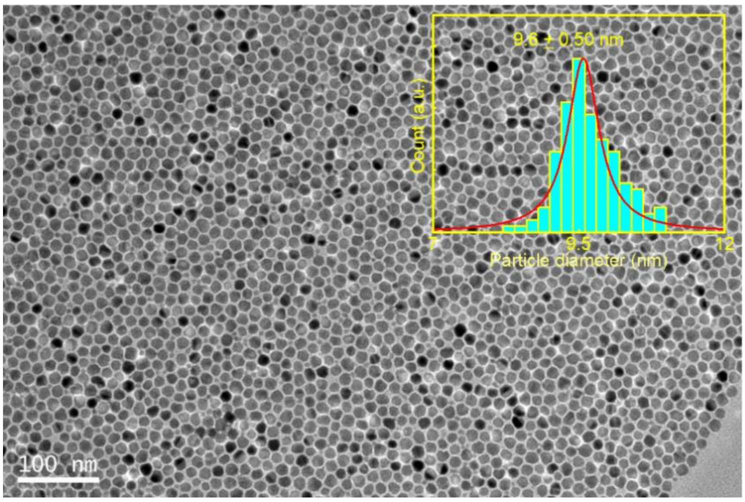


**Figure S2. TEM image of monodisperse HgTe CQDs.** Inset shows the size distribution of the HgTe CQDs with a mean size 9.6 nm.

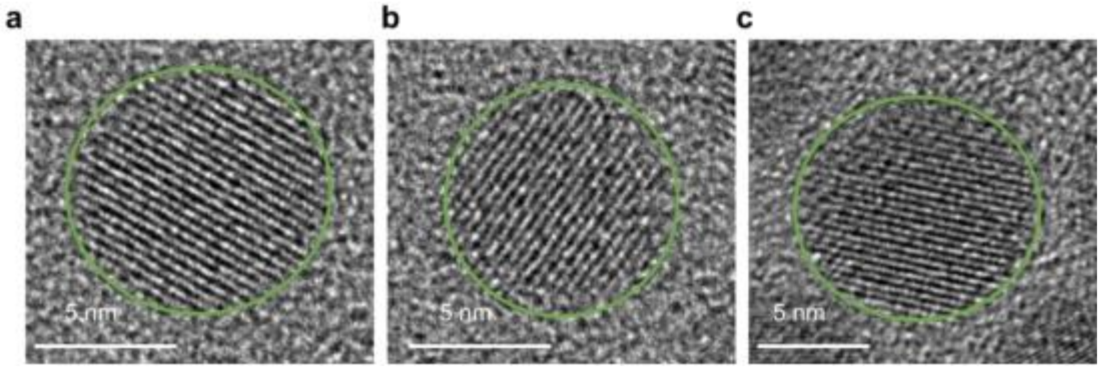


**Figure S3. High resolution TEM images of HgTe CQDs. a-c**) Green outline indicates edges, highlighting approximate-uniform shapes.

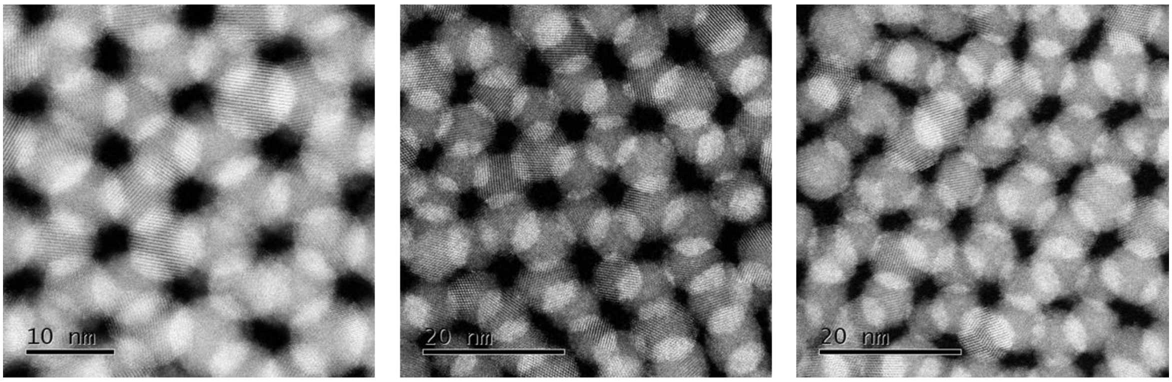


**Figure S4. More HAADF-STEM images of CQDs' honeycomb structures.**

*Detailed blade coating methods.*

Currently, the commonly used deposition techniques for colloidal quantum dots are layer-by-layer spin-coating and spray-coating. However, in the case of spin-coating, the amount of wasted CQDs is enormous and more importantly, those wasted HgTe CQDs are highly toxic. In the case of spray-coating, the fabricated films usually shown a porous structure with strikingly non-uniform morphologies and insulting organic ligand aggregation, both of which is not ideal for photo-detection applications[2]. As is suggested by Ted Sargent[3], blade coating (Fig. S5) shown superiority in film quality and film thickness, and it is considered as a scalable,

industrial-level technique, which is closely related to roll-to-roll printing. Blade coating entails the spreading of the solution over the substrate by a blade. In our work, we place a solution of quantum dots on a heated (45°C) substrate and start a mechanical device to carry the blades at a certain speed of directional movement, which will form a homogeneous film of quantum dots. By taking different amounts of the quantum dot solution, films can be prepared from order of hundreds of nanometers to a few microns thick as shown in Fig. S6. And the SEM images of the quality of the films prepared by scratch deposition are shown in Fig.S7.

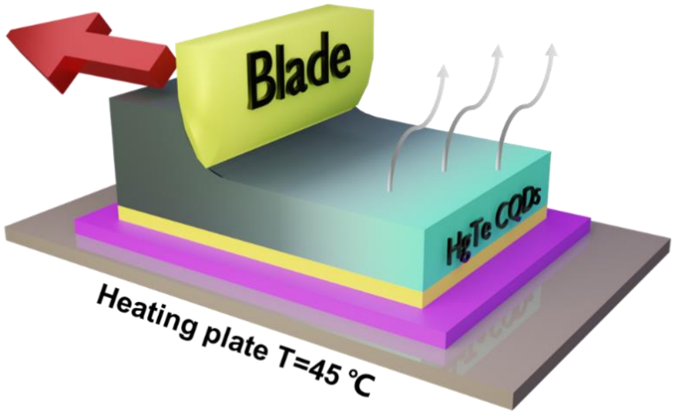


**Figure S5. Scheme of the blade-coating process.**

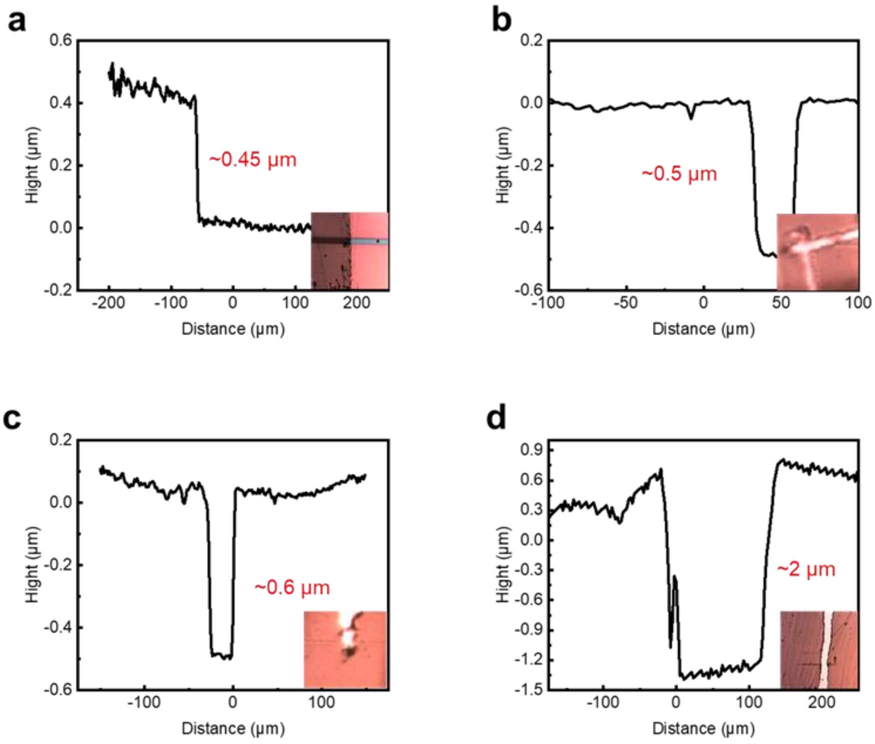


**Figure S6. Thickness of the blade-coated films from HgTe CQDs inks.**

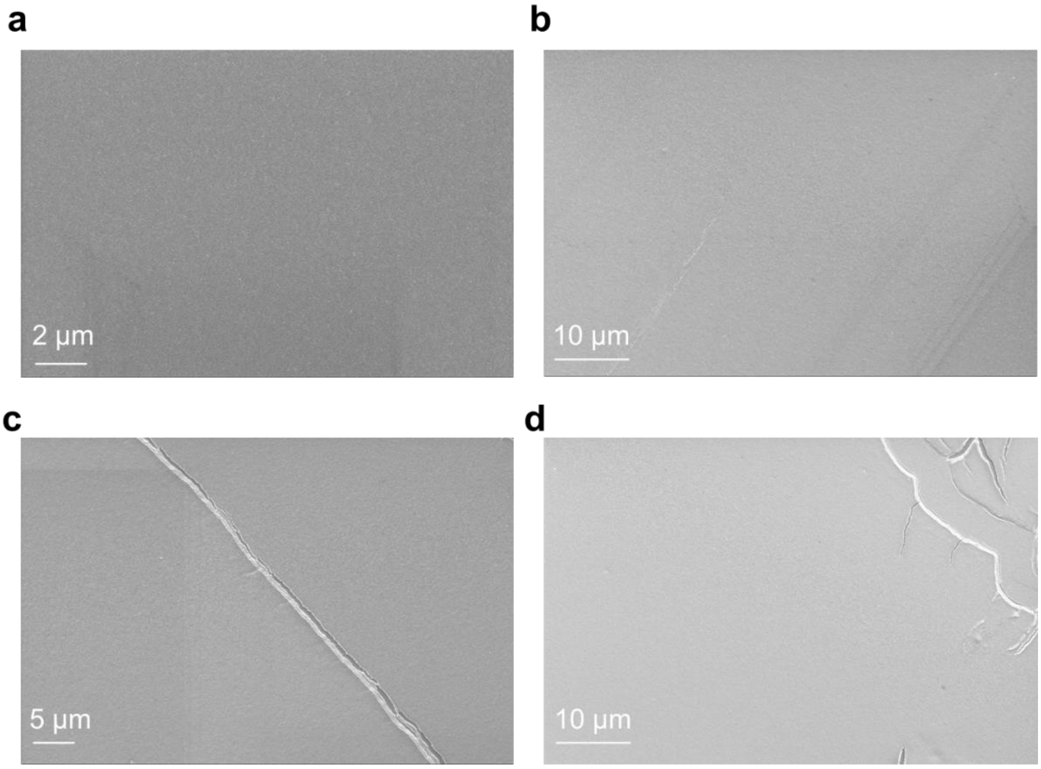


**Figure S7. SEM images of HgTe CQDs film.**

*Flat and smooth optical absorption spectra.*

One possible explanation for wavelength independent responsivity could be higher optical absorption at lower wavelength, and by coincidence, the increased amount of absorption perfectly countered the less conversion efficiency. However, through infrared optical transmission measurements, a smooth and near unity absorption profile is observed in our fabricated HgTe superlattice films, prior to the onset of cutoff wavelength without the presence of any excitonic absorption peaks, shown in Fig. S8.

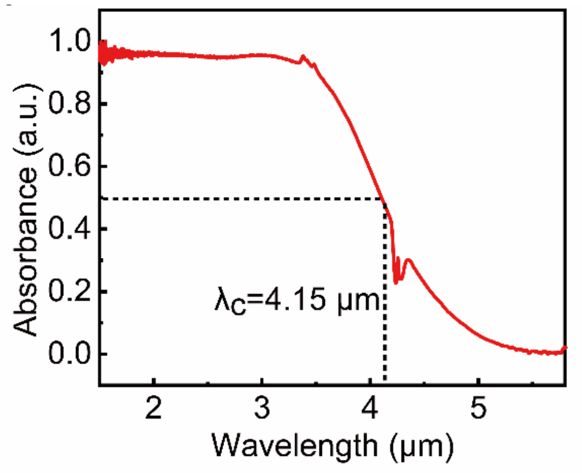


**Figure S8 Infrared absorption spectra of HgTe CQDs.** The cutoff wavelength is ~4.15 μm.

*Photoconductive response in Conventional HgTe CQDs.*

Devices prepared using quantum dots synthesized through the conventional hot-injection method exhibit a typical photoconductive effect, as illustrated in Fgi. S9. Likely, with conventional synthesis, due to the absence of long-range periodicity or topologically nanogeometry, hot-carriers generate and transport in classical free charge carriers' model.

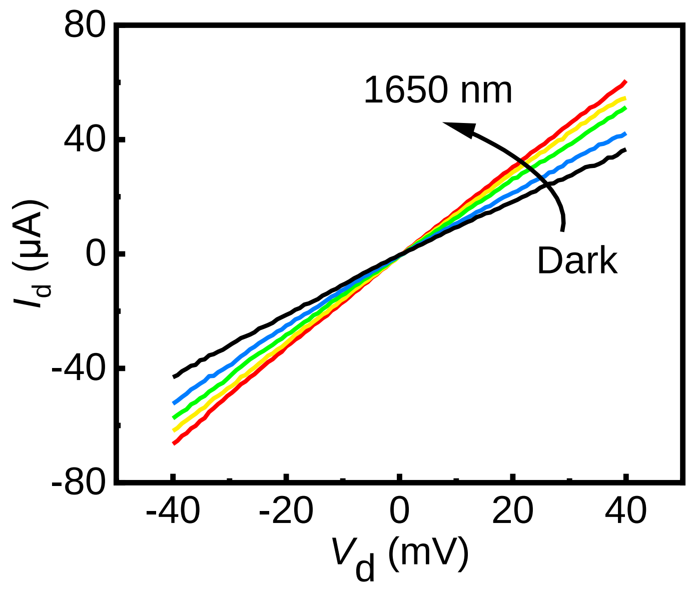


**Figure S9. A typical photoconductive response based on conventional synthesized HgTe CQDs films.**

<u>*Seebeck measurements.*</u>

The Seebeck effect is a phenomenon that occurs in thermoelectric materials. In P-type semiconductors, by introducing a controlled temperature gradient to the material, due to the high concentration of holes at the hot end, the holes will spread from the high temperature end to the low temperature end. Therefore, an electric field appears inside the semiconductor and then caused a voltage difference, known as the thermoelectric voltage or Seebeck voltage.

Here, through NETZSCH's SBA 458 Nemesis, the obtained Seebeck coefficient of our HgTe CQDs superlattice is around 400 μV $K^{-1}$ (Fig. S10), which is significantly higher than those measured using alternative nanocrystals (NCs) thin films and pellents. For example, S = -140 μV $K^{-1}$ for $Bi_2Te_3$ NCs[4], S = -100 μV $K^{-1}$ for $Ag_2Te$ NCs[5], S = 20 μV $K^{-1}$ for $FeSb_2$ NCs[6], $S_{max}$ = -35 μV $K^{-1}$ for HgTe CQDs[7].

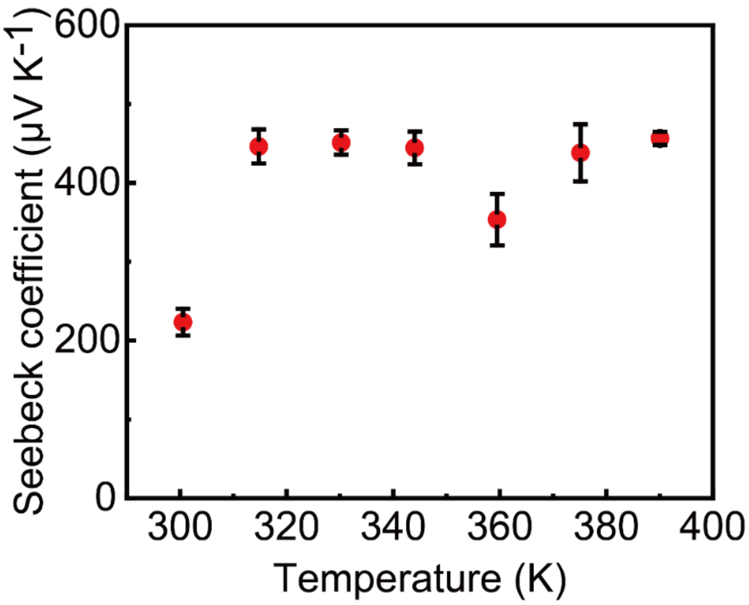


**Figure S10. Seebeck coefficient of HgTe QDs.** The positive coefficient reveals the p-type nature of HgTe CQDs.

*Thickness dependent hot-carrier assisted photocurrent.*

As is mentioned in the main-text, with limited carrier mobility and fast recombination time, the light-induced photocurrent would quickly saturate over hundreds of nanometers, commonly observed in colloidal quantum dots systems[8]. However, in our experiments, we clearly observed increased photoresponse in thicker CQDs films. As is illustrated in Fig. S11 and S12, in our thickest CQDs films (~750 nm), the highest photoresponse was observed. Therefore, it is reasonable to believe that the mean free path (products of hot-carrier cooling time and charge carrier's mobility) is quite a bit longer than 750 nm.

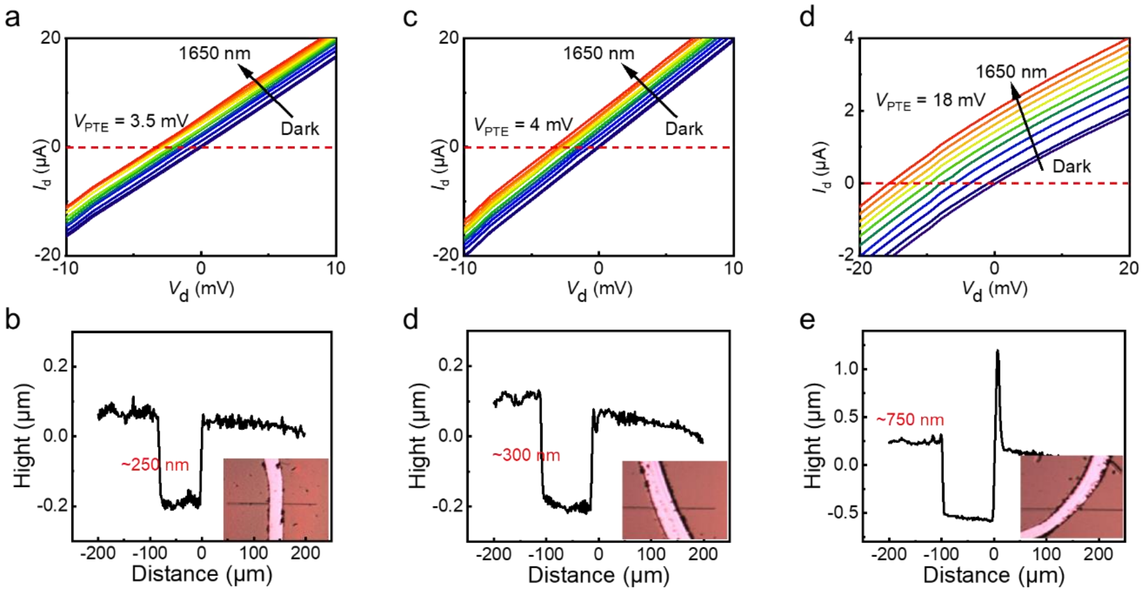


**Figure S11. Photothermoelectric voltage at different thicknesses.**

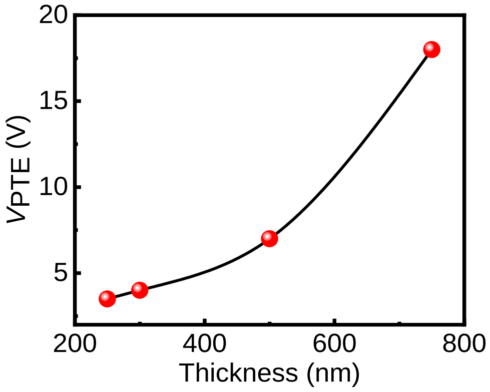


**Figure S12. $V_{PTE}$ versus the thickness of the HgTe CQDs film.**

*Discussion about Schottky junction near the edge of gold electrodes.*

To better understand the light-induced photocurrent at zero bias and exclude the possible influence from Schottky junction, we embark on a brief exploration of the scenario that emerges when a conventional metal-semiconductor contact.

Given that the doping concentration within the quantum dot is considerably lower than that of gold, an asymmetric abrupt junction takes shape, resulting in the depletion layer of the junction being primarily situated within the confines of the quantum dot.

This configuration aligns with the characteristics of a unilateral abrupt junction, as per the context of the study.

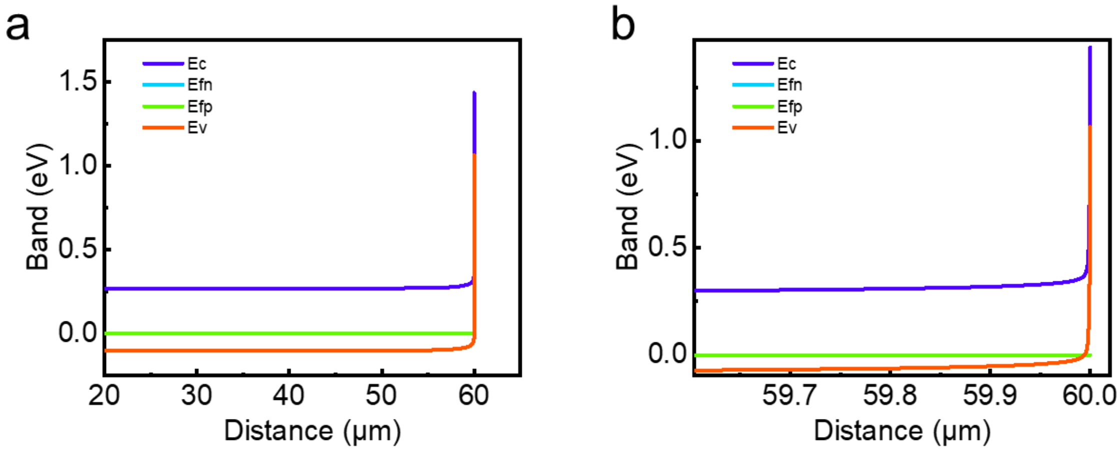


**Figure S13. TCAD results of band diagram of HgTe CQD and Au electrodes.**

The depletion layer width of this unilateral abrupt junction can be calculated by formula: $W = \left(\frac{2\varepsilon\Psi_0}{qN_d}\right)^{\frac{1}{2}}$, where $W$ is depletion layer width, $\varepsilon$ is the dielectric constant, $\psi_0$ is the built-in potential difference between the quantum dots and gold upon contact, $q$ is the fundamental charge that the value is $1.602 \times 10^{-19}$ coulombs, $N_d$ is the doping concentration. For the HgTe CQD we used, $\varepsilon_{CQD}$ is taken as 15.1, $\psi_0$ is taken as 1.4 V, $N_d$ is taken as $1.08 \times 10^{-20}$ $cm^{-3}$. Therefore, by calculation, the depletion layer width of the Schottky junction formed in our device is 4.84 nm. Meanwhile, we also performed simulation calculations using TCAD software and obtained the schematic diagram of the energy band of the quantum dot-gold contact, as shown in Fig. S13, that the depletion layer width of the Schottky junction is indeed on the order of nanometers. Thus, in operation under zero bias voltage, the diffusion of current remains confined to this nanoscale extent.

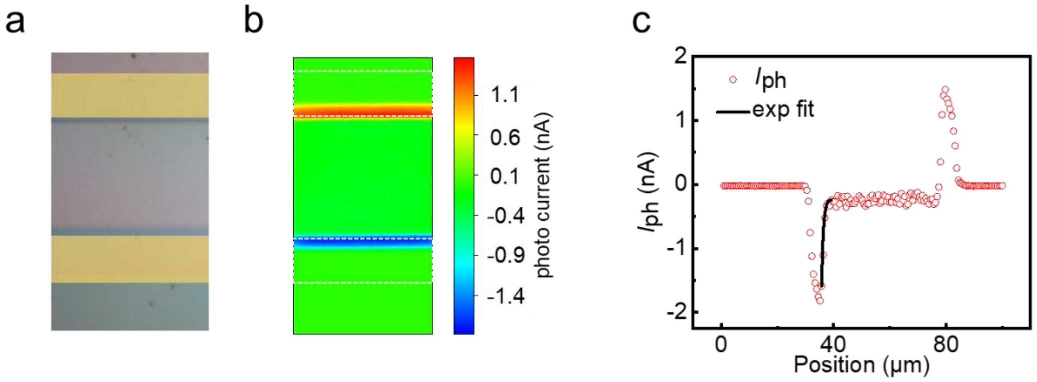


**Figure S14. Photocurrent mapping measured at zero bias in 1650-nm laser illumination based on conventional synthesized HgTe CQDs.**

To better confirm that Schottky junction only existed at the edge of gold electrodes, we performed 2D raster scanned photocurrent mappings under illumination of a 1650-nm laser at zero bias based on the conventional synthesized CQDs. As expected, the results of photocurrent mapping (Fig. S14) indicated that the photodetector exhibits a photo-active area almost fully-confined in gold electrodes and the extracted diffusion length was about hundreds of nanometers, which is majorly originated from interface Schottky junction.

*Simulation of low-angle XRD periodic peaks.*

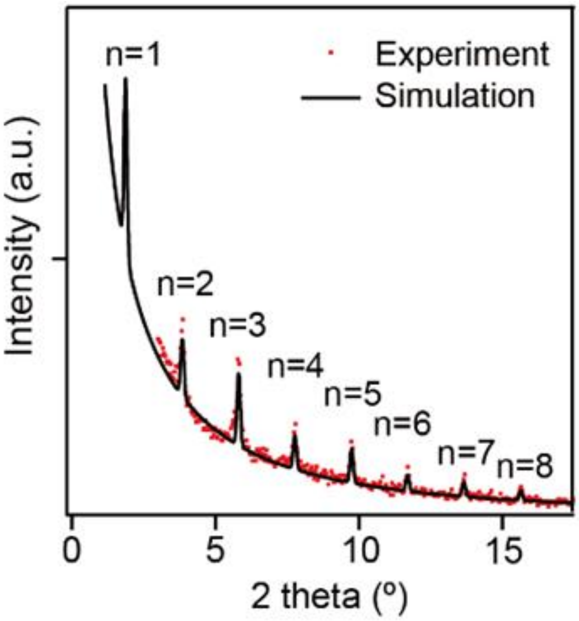


**Figure S15. Experimental (red circle) and calculated (black line) low-angle XRD profile for the HgTe CQDs superlattice.**

Fullerton et al. present a comprehensive method for precisely refining the intricate structural aspects of superlattice configurations[9]. To comprehensively analyze a wide range of superlattices, they devised a versatile kinematical diffraction equation that encompasses structural variations—be they random, continuous, or discrete—in relation to the average composition. At its core, the low-angle diffraction profile theoretically offers a direct depiction of the Fourier transform of the compositional profile. However, challenges arise due to disorder, multiple reflections (necessitating dynamical corrections), refraction phenomena, and surface reflections, which collectively constrain the insights attainable from a Fourier transform analysis of the diffraction profile.

A prominently employed technique to compute the low-angle profile entails iteratively applying optical theories. In this approach, the layers are modeled as a continuous medium, and computations are executed for the reflections transpiring at each interface. These theories have been verified to be equivalent to dynamic calculations, encapsulating aspects like absorption, refraction, surface, and substrate reflections. Furthermore, these models can be flexibly adapted to account for the

effects of interdiffusion.

$$I_c(q) = I(q)S\left[\frac{\left(1-e^{-\frac{2\mu\tau}{sin\theta}}\right)(1+cos^2 2\theta_m cos^2 2\theta)}{sin2\theta}\right] + I_b$$

where S is a scaling factor, μ is the average absorption coefficient, τ the total thickness of the superlattice, $2\theta_m$ the scattering angle of the monochromator, and $I_b$ is the background intensity. Here, $I(q)S$ is 40.81, $2\mu\tau$ is 0.1737, $\theta_m$ is 0.2913 rad, $I_b$ is 33.78.

And the position of the peaks in the low-angle region is given by

$$sin^2\theta = (\frac{n\lambda_x}{2\Lambda})^2 + 2\delta_s,$$

where θ is the angle of the peak position, n is the order of reflection, $\lambda_x$ is the x-ray wavelength, here is Cu-K alpha rays with the wavelength of 1.54Å, $\delta_s$ is typically ≈ $3 \times 10^{-5}$, Λ is about 2 nm.

Employing this model as a foundation, we conducted meticulous numerical fitting and computations, revealing a remarkable congruence between the calculated values and experimental data. Furthermore, by extending the model to encompass θ values up to 1°, we extrapolated and theoretically predicted the position and intensity of the initial diffraction peak. This predictive illustration is presented in the accompanying Fig. S15.

*Discussion about existence of long-range periodicity over a scale larger than micrometers.*

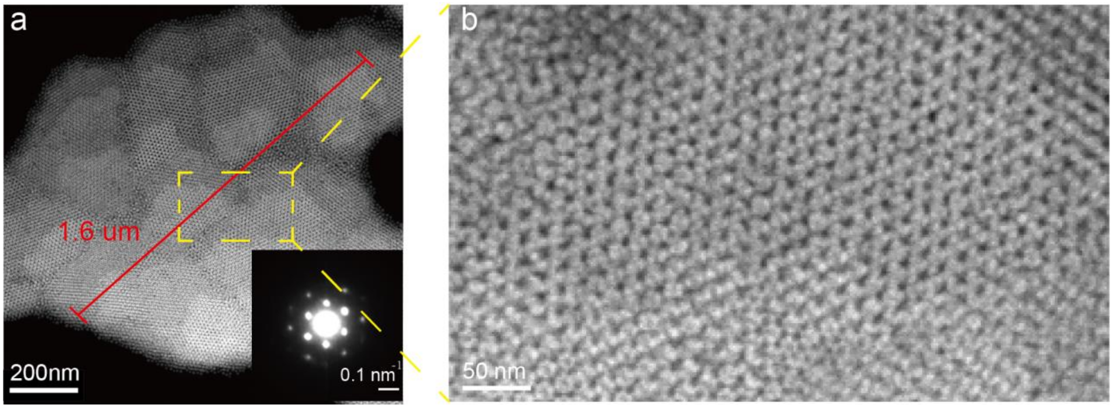


Figure S16. TEM images of HgTe quantum dots superlattice. a) HR-TEM image of micro-meter HgTe quantum dots pack. Inset: corresponding SAED images, scale bar: 0.1 nm-1 b) Zoom-in pictures from yellow-line highlighted area in a).

In the maintext, we have mentioned the estimation of long-range periodicity over a scale larger than micrometers, due to the following rationales:

Firstly, the presence of 1 μm long-range periodicity was actually directly observed through high-resolution transmission electron microscopy (HR-TEM) in Fig. 1a. Here, as presented in Fig. S16, a zoomed-in view of the yellow selected area from Fig. 1a clearly reveals the honeycomb structures. This observed periodicity extends for approximately 1.6 μm.

Additionally, an alternative approach for determining the dimensions of the proposed honeycomb lattice involved the analysis of X-ray diffraction (XRD) patterns using the well-established Scherrer equation. The Scherrer equation relates the size of sub-micrometer crystallites to the broadening of a diffraction peak. In this case, the equation is defined as follows:

$$D = K\lambda / (\beta \cos \theta)$$

where:

D is the average crystallite size (grain size),

K is the Scherrer constant (typically taken as 0.9),

λ is the wavelength of the X-ray or neutron radiation,

$\beta$ is the full width at half maximum (FWHM) of the diffraction peak (in this particular case, a value of 0.087 was measured)

$\theta$ is the Bragg angle (2theta = 3.8).

By applying the Scherrer equation to the XRD data obtained from Fig. 1g, 1h, and Supplementary Fig. S15, a grain size of 95.46 nm was determined. However, it is important to note that the Scherrer equation is primarily applicable to nano-scale crystallites, and the obtained grain sizes, or more precisely, the coherently scattering domain size, which can be smaller than the crystallite size. Consequently, it is crucial to acknowledge that the Scherrer equation provides a conservative estimation of the lower limit for the coherently scattering domain size. Therefore, the observed grain size of 95.46 nm is indicative of the presence of long-range periodicity, at the very least, within a micrometer-scale region.

The combination of direct observation using HR-TEM and estimation based on XRD analysis provides substantial evidence to support the assertion that the proposed honeycomb lattice in CQD films exists on a micrometer scale.

*Blackbody photoresponse and noise measurement and analysis.*

Certainly, noise measurement is crucial for evaluating a detector as it directly impacts the device's performance and sensitivity. In our study, we conducted noise spectrum measurements on the device, as illustrated in Fig. S17. Initially, the sample was encapsulated in Dewar and evacuated to create a vacuum environment. Subsequently, the current signal from the device was converted to a voltage signal using a pre-amplifier (Stanford Research Systems SR570) and then output to a spectrum analyzer (Keysight 35670A Dynamic Signal Analyzer) for data acquisition. Based on the noise spectrum, we calculate and plot the blackbody responsivity R and detectivity $D^*$ against the power of blackbody as shown in Fig. S18b. The detailed calculation process is shown below. The total incident power on the device surface

can be calculated by the approximation formula $P=\frac{\alpha\varepsilon\sigma(T^4-T_0^4)AB^2}{\pi L^2}$, where α is the modulation factor, ε is the average emissivity of the blackbody source, ε is 0.9 for our blackbody source, σ is the Stefan-Boltzmann constant. T and $T_0$ are the temperature of the blackbody radiation source and the room temperature (300 K), respectively. A is the device area in units of square centimeters. B is the aperture radius of the blackbody radiation source, here is 0.4 inch, and L is the distance between the aperture and sample in units of centimeters. And the responsivity of blackbody can be calculated by the formula $R_{bb}=\frac{V_s}{P}$, where $V_s$ is the measured signal voltage. Then we can calculate the detectivity of blackbody by the formula $D_{bb}^*=\frac{R_{bb}}{V_n}\sqrt{A\bullet\Delta f}$, where $V_n$ is the measured noise voltage, Δf is the bandwidth of spectrum analyzer. In the description here, $D_{bb}^*$ refers to $D^*$ in the main text. And the unit of $R_{bb}$ is V/W, we convert that to the unit of A/W. The responsivity of blackbody with the unit of A/W can be calculated by the formula $R=\frac{V_s}{SP}$, where S is the magnification of the preamp in our measurement.

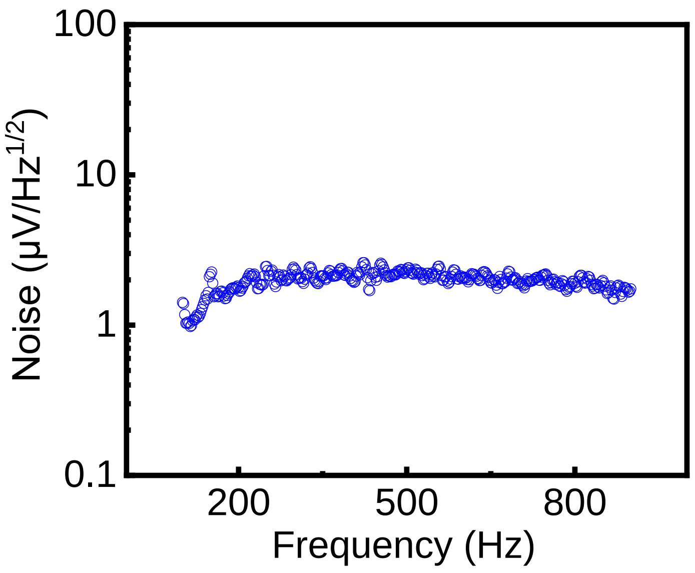

**Figure S17. Noise spectrum measured at background temperature of 300 K and zero bias.**

*Discussion about the potential to be used as a photodetector.*

To highlight the photodetection capabilities of the HgTe quantum dots (QDs) photodetector, we employed the device depicted in Fig. 2d of the main text. The results of our experimentation are presented in Fig. S18, showcasing the device's performance in capturing blackbody radiation and reflecting images. **Fig.** S18a illustrated the setup of blackbody detection system. The photoresponse was clearly observed in our detectors under the illumination of a calibrated blackbody source at 1073 K, chopped at 500 Hz. As is shown in **Fig.** S18b, we have plotted the blackbody responsivity $R$ and detectivity $D^*$ against the power of blackbody. The $R$ and $D^*$ of our PTE devices reached up to 1.22 mA $W^{-1}$ and $1.06 \times 10^7$ cm $Hz^{-1/2}$ $W^{-1}$, respectively. And a reflection imaging system has been set up, as shown in **Fig.** S18c. A logo of Shanghai Institute of Technical Physics (SITP) mask was prepared as a test object. The image of a 2D raster scan was acquire by the detector, with total $160 \times 100$ points acquired by lock-in technique. The imaging results are shown in **Fig.** S18d. The obtained image shown good quality that even some defects of the mask could be distinguished, indicating that our detector was indeed feasible for imaging application. This visualization underscores the potential and efficacy of the photodetector in practical applications, demonstrating its ability to detect and respond to incident radiation.

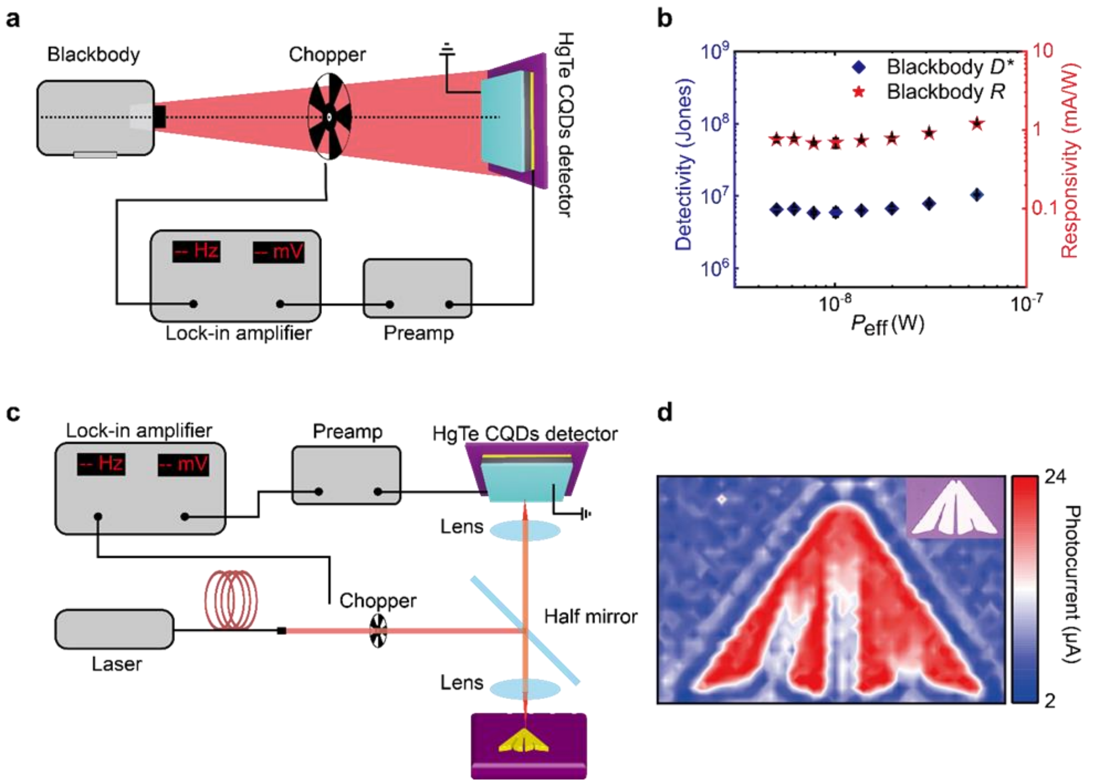


**Figure S18. Blackbody detection and reflecting images of photodetector based HgTe CQDs.** a) Schematic of the setup of the system of the measurements of the blackbody response. b) Blackbody responsivity and detectivity of HgTe CQDs detector versus the illumination power of blackbody. c) Schematic of the reflection imaging systems for HgTe CQDs photodetector. d) Imaging by HgTe CQDs photodetector under the illumination of reflection of 520 nm laser.

**Table S1. Comparison of thermal carrier transport distances in different material systems.**

| Materials | Transport distanc | Reference |
|---|---|---|
| $CH_3NH_3PbI_3$ QD | 230 and 600 nm | [10] |
| $MAPbI_3$ | 150 nm | [11] |
| Pentacene | 32 nm | [12] |
| Si | ~ Nanometer scale | [13] |

| | | |
|---|---|---|
| $WS_2$ | 500 nm | 14 |
| Cu/GaN | ~20 - 30 nm | 15 |
| $Cs_2AgBiBr_6$ | > 200 nm | 16 |
| HgTe QD | ~15 μm | This work |

Reference.


1 Shen, G., Chen, M. & Guyot-Sionnest, P. Synthesis of Nonaggregating HgTe Colloidal Quantum Dots and the Emergence of Air-Stable n-Doping. *J. Phys. Chem. Lett.* **8**, 2224-2228 (2017).

2 Zhao, Q. *et al.* Colloidal Quantum Dot Solar Cells: Progressive Deposition Techniques and Future Prospects on Large-Area Fabrication. *Adv. Mater.* **34**, 2107888 (2022).

3 Fan, J. Z. *et al.* Micron thick colloidal quantum dot solids. *Nano letters* **20**, 5284-5291 (2020).

4 Son, J. S. *et al.* n-Type nanostructured thermoelectric materials prepared from chemically synthesized ultrathin Bi2Te3 nanoplates. *Nano Lett* **12**, 640-647 (2012).

5 Zhou, W. *et al.* Preparation and thermoelectric properties of sulfur doped Ag2Te nanoparticles via solvothermal methods. *Nanoscale* **4**, 3926-3931 (2012).

6 Ko, D. K., Urban, J. J. & Murray, C. B. Carrier distribution and dynamics of nanocrystal solids doped with artificial atoms. *Nano Lett* **10**, 1842-1847 (2010).

7 Lan, X. *et al.* Quantum dot solids showing state-resolved band-like transport. *Nat Mater* **19**, 323-329 (2020).

8 Istva´n Robel, V. S., Masaru Kuno, and Prashant V. Kamat. Quantum Dot Solar Cells. Harvesting Light Energy with CdSe Nanocrystals Molecularly Linked to Mesoscopic TiO2 Films. *J. Am. Chem. Soc.* (2005).

9 Fullerton, E. E., Schuller, I. K., Vanderstraeten, H. & Bruynseraede, Y. Structural refinement of superlattices from x-ray diffraction. *Phys Rev B Condens Matter* **45**, 9292-9310 (1992).

10 Zhi Guo, Y. W., Mengjin Yang, Jordan Snaider, Kai Zhu, Libai Huang. Long-range hot-carrier transport in hybrid perovskites visualized by ultrafast microscopy. *Science* (2017).

11 Sung, J. *et al.* Long-range ballistic propagation of carriers in methylammonium lead iodide perovskite thin films. *Nat. Phys.* **16**, 171-176 (2019).

12 Schnedermann, C. *et al.* Ultrafast Tracking of Exciton and Charge Carrier Transport in Optoelectronic Materials on the Nanometer Scale. *J. Phys. Chem. Lett.* **10**, 6727-6733 (2019).

13 Ge, J., Luo, M., Zou, W., Peng, W. & Duan, H. Plasmonic photodetectors based on asymmetric nanogap electrodes. *Appl. Phys. Express* **9** (2016).

14 Liu, Q. *et al.* Visualizing Hot-Carrier Expansion and Cascaded Transport in WS(2) by Ultrafast Transient Absorption Microscopy. *Adv Sci (Weinh)* **9**, e2105746 (2022).

15 Tagliabue, G., DuChene, J. S., Habib, A., Sundararaman, R. & Atwater, H. A. Hot-Hole versus Hot-Electron Transport at Cu/GaN Heterojunction Interfaces. *ACS Nano* **14**, 5788-5797 (2020).

16 Heng Zhang, E. D., Wenhao Zheng, Shuai Fu, Lucia D. Virgilio, Pushpendra Kumar1, Mischa Bonn, Hai I. Wang. Highly mobile hot holes in Cs2AgBiBr6 double perovskite. *Sci. Adv.* (2021).
